# Structure characterization and anticoagulant activity of a novel polysaccharide from *Leonurus artemisia* (Laur.) S. Y. Hu F

**DOI:** 10.1039/c9ra10853j

**Published:** 2020-01-10

**Authors:** Cheng Hu, Hao-Xuan Li, Meng-Ting Zhang, Li-Fang Liu

**Affiliations:** State Key Laboratory of Natural Medicines, Department of Chinese Medicines Analysis, School of Traditional Chinese Pharmacy, China Pharmaceutical University No. 24 Tongjia Lane Nanjing 21198 China liulifan69@126.com +86 25 8618 5136 +86 25 8618 5136

## Abstract

An acidic polysaccharide, named LAP-1, was extracted and isolated from *Leonurus artemisia* (Laur.), and was further purified with ion exchange chromatography and gel chromatography. The extraction conditions of the crude polysaccharides were optimized by single-factor experiments and response surface methodology. The primary structure of the purified polysaccharide was measured by FT-IR, GC-MS, and NMR. The results showed that LAP-1 was mainly composed of galacturonic acid (GalA), mannose (Man), xylose (Xyl), rhamnose (Rha), arabinose (Ara), glucose (Glc), galactose (Gal), fucose (Fuc), ribose (Rib), and glucuronic acid (GlcA) in the molar ratio of 8.74 : 3.45 : 1.02 : 1 : 2.11 : 5.60 : 4.73 : 1.08 : 1.09 : 1.47. Primary structure analysis results indicated that LAP-1 contained characteristic glycosyl linkages such as →1)-α-d-Man*p*, →1)-α-d-Glc*p*, →1)-α-d-Ara*p*-(2→, →1)-β-d-Gal*p*-(3→, →1)-β-d-Man*p*-(4→, →1)-β-d-Gal*p*-(4→, →1)-β-d-Glc*p*-(4→, →1)-β-d-GalA*p*-(4→, →1)-β-d-GlcA*p*-(4→, →1)-β-d-Man*p*-(4,6→, →1)-β-d-Man*p*-(3,4→. The *M*_w_/*M*_n_ (PDI), *M*_n_, *M*_*z*_ and *M*_w_ of LAP-1 were determined to be 1.423, 6.979 × 10^3^ g mol^−1^, 1.409 × 10^4^ g mol^−1^, and 9.930 × 10^3^ g mol^−1^ by HPSEC-MALLS-RID and DLS. SEM, TEM and AFM results indicated that LAP-1 was a highly branched structure. LAP-1 showed mild anticoagulant activity, low toxicity, and less spontaneous bleeding compared with heparin sodium. These results demonstrated the effective coagulation activity of *Leonurus artemisia* polysaccharides. Thus, the purified LAP-1 could be explored as a promising anticoagulant agent for the treatment of coagulation disorders.

## Abstract 2

An acidic polysaccharide, denoted LAP-1 was extracted, isolated and purified from *Leonurus artemisia* (Laur.), in addition to its structure and anticoagulant activity were explored.

## Graphical abstract

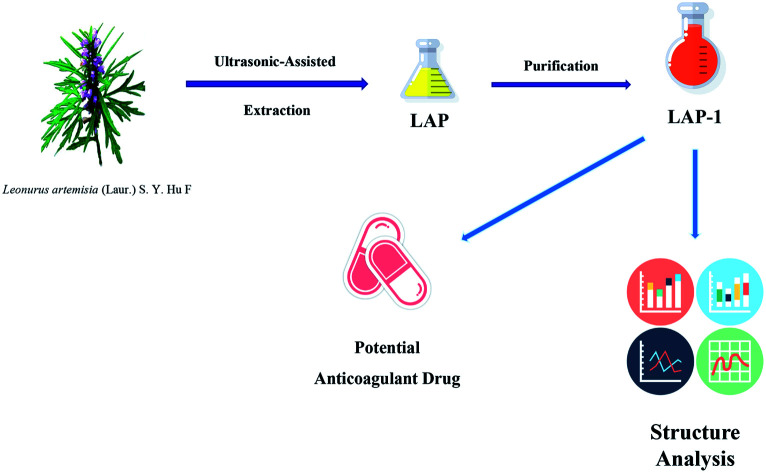

## 1. Introduction

Leonuri herba, also called motherwort herb in China, is a famous traditional Chinese medicine (TCM) obtained from the fresh or dried aerial part of *Leonurus artemisia* (Laur.) S. Y. Hu F. As recorded in the Chinese Pharmacopoeia (2015 edition), it has been a commonly used herb for treating irregular menstruation, dysmenorrhea, amenorrhea, lochia, edema of acute nephritis and blood system diseases.^1^ Recent studies have also demonstrated its outstanding functions in promoting blood circulation and regulating menstruation, diuresis and detumescence.^2^

*Leonurus artemisia* contains many chemical constituents with a variety of pharmacological effects, such as alkaloids for antioxygenation, diterpenoids for anti-platelet agglutinating, flavonoids for uterine excitation and volatile oil for bacteriostasis.^3^ Most of the previous literature has attributed its anticoagulant activity to its small molecule components such as leonurine, genkwanin, and aethylparabenum.

To date, there has been no report about the relationship between its anticoagulant activity and polysaccharides in *Leonurus artemisia*. Nevertheless, the anticoagulant function of herbal polysaccharides should not be ignored, especially since most of the archaic TCM decoctions were prepared *via* water boiling. More recently, several herbal polysaccharides have been demonstrated to have significant anticoagulant activity.^4^ However, as one of the abundant components in *Leonurus artemisia*, the anticoagulant effect of *Leonurus artemisia* polysaccharides has seldom been reported. As is well known, the structural properties of polysaccharides such as ratios of constituent monosaccharides,^5^ molecular size, types and chain conformations, and features of glycosidic linkages are closely correlated to their bioactivities.^6,7^

Therefore, this work aimed to extract, purify and investigate the fundamental structure information, such as the chemical properties of polysaccharides from *Leonurus artemisia*, in addition to examining its anticoagulant activity.

## 2. Materials and methods

### 2.1 Materials and reagents

*Leonurus artemisia* samples were obtained from Jiangxi Xinzheng Pharmaceutical Co., Ltd., Pingxiang, China. The sephacryl S-200 and diethylaminoethylcellulose-52 (DEAE-52) were obtained from Whatman International, Ltd. (Kent, UK). Ethanol, sulfuric acid, hydrochloric acid, phenol, ethyl ether and other reagents were purchased from the Nanjing Chemical Reagent Co., Ltd. (Nanjing, China). All of the chemicals reagents and chemicals were of analytical grade.

### 2.2 Ultrasonic-assisted extraction of *Leonurus artemisia* polysaccharide

The dried aerial part of the *Leonurus artemisia* samples were ground and filtered through an 80 mesh. Ethanol was used for defatting and decoloring at 60 °C for 3 h in a Soxhlet extractor system and then centrifuged at 8000 rpm for 15 min. The precipitate was dried through vacuum dehydration. Then, the ultrasonic-assisted extraction of the polysaccharides was performed in an ultrasonic cleaner at 40 kHz (KH-250DV, Kunshan Hachuang Ultrasonic Instrument Co., Ltd., Kunshan, China).

#### 2.2.1 Single factor analysis

Four major factors (water-to-raw material ratio, extraction time, extraction temperature, ultrasonic power) were selected for further study.^8^ The effects of each factor on the yields of LAP were measured through single factor experiments. Pretreated samples (10 g) were prepared in a specific water-to-raw material ratio (20 to 70 mL g^−1^), extraction time (20 to 70 min), ultrasonic power (125 to 250 W), and extraction temperature (20 to 70 °C). The supernatant was collected after centrifugation at 8000 rpm for 15 min, and then the polysaccharides components were precipitated by 95% ethanol. After 24 h precipitation, the precipitate was collected after centrifugation at 8000 rpm for 15 min. The sugar content of the crude polysaccharides was detected by the phenol–sulfuric acid method.^9^ Under the conditions that only one factor changed while others stayed constant, each experiment was carried out. The effect of the four factors were evaluated by the yields of LAP. The extraction yield of LAP was calculated with the following formula:

1 Extraction yield (%) = (*S* × *W*_1_/*W*_0_) × 100

*S*: sugar content of crude polysaccharides, *W*_1_: weight of crude polysaccharides, *W*_0_: weight of powdered *Leonurus artemisia*.

#### 2.2.2 Box–Behnken design (BBD)

Box–Behnken design with four independent variables (*A*, extraction time; *B*, extraction power; *C*, extraction temperature; *D*, ratio of water to material) at three levels (−1, 0, +1) was performed. The following formula for statistical calculation was used to code the above values.2
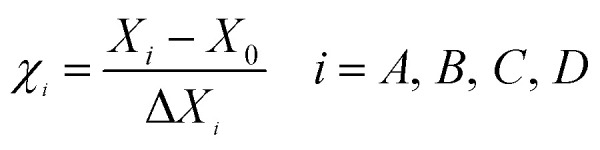
*X*_0_: actual value of the *X*_*i*_ on the center point, *X*_*i*_: actual value of the variable, Δ*X*_*i*_: step change value, *χ*_*i*_: coded value of the variable.

The experimental runs and the values of the four independent variables for BBD are showed in Table 1. The interrelationships and relationships between the independent variables and the response are indicated in the second-order polynomial model (eqn (3)) for predicting the optimized conditions.3
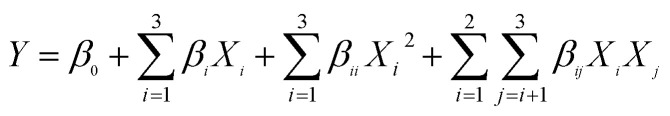
*β*_*i*_: regression coefficients for the linear, *β*_0_: regression coefficients for the intercept, *β*_*ij*_: regression coefficients for the interaction, *β*_*ii*_: regression coefficients for the quadratic, *X*_*i*_: coded values of independent variables, *X*_*j*_: coded values of independent variables, *Y*: predicted response.

**Table 1:** Box–Behnken experimental design and the results for LAP yield of ultrasound-assisted extraction (*n* = 3)

Run	Coded variable levels				Yield of LAP (%)
	Extraction time (min)	Extraction power (W)	Extraction temperature (°C)	Ratio of water to material (mL g^−1^)	
	Factor 1: *A*	Factor 2: *B*	Factor 3: *C*	Factor 4: *D*	
1	−1	0	−1	0	2.16 ± 0.19
2	−1	0	0	1	2.30 ± 0.55
3	1	0	0	−1	2.06 ± 0.22
4	0	0	1	−1	2.15 ± 0.36
5	0	0	0	0	3.35 ± 0.11
6	0	1	−1	0	2.75 ± 0.24
7	1	−1	0	0	2.50 ± 0.15
8	−1	1	0	0	2.79 ± 0.42
9	0	−1	−1	0	2.72 ± 0.07
10	0	−1	0	−1	2.05 ± 0.19
11	0	0	0	0	3.29 ± 0.19
12	−1	−1	0	0	2.26 ± 0.38
13	1	1	0	0	2.67 ± 0.22
14	0	1	1	0	3.12 ± 0.23
15	0	−1	1	0	2.85 ± 0.11
16	−1	0	0	−1	1.72 ± 0.46
17	0	1	0	−1	2.29 ± 0.06
18	0	−1	0	1	2.86 ± 0.37
19	0	0	1	1	2.73 ± 0.09
20	0	0	0	0	3.23 ± 0.09
21	−1	0	1	0	2.66 ± 0.22
22	0	0	0	0	3.34 ± 0.34
23	1	0	0	1	2.27 ± 0.17
24	1	0	1	0	2.56 ± 0.09
25	0	0	−1	−1	2.10 ± 0.58
26	1	0	−1	0	2.49 ± 0.09
27	0	1	0	1	2.96 ± 0.21
28	0	0	−1	1	2.76 ± 0.25
29	0	0	0	0	3.30 ± 0.29

The related experimental data were calculated and analyzed by Design-Expert software (version 11.0, Stat-Ease Inc., Minneapolis, USA). The validity of the statistical experimental design was verified by confirmation experiments under the optimized conditions. The LAP samples obtained from the optimized conditions were used for further purification steps.

### 2.3 Purification of polysaccharide from crude *Leonurus artemisia* polysaccharides

*Leonurus artemisia* polysaccharides (LAP) was first deproteinated and depigmented by macroporous adsorptive resin D101. Then, further deproteinization was performed with the Sevag method 5 times, followed by dialysis (cut-off *M*_w_ 3500 Da) against distilled water for 72 h. The polysaccharides components of the dialysates were precipitated by ethanol. The precipitate was sequentially washed with ethanol, acetone and ether, followed by freeze-drying. Ion-exchange chromatography (DEAE-32) with a gradient of 0–2.0 mol L^−1^ NaCl and a Sephacryl S-200 column were then used to purify the obtained sample. During the purification process, an acid polysaccharide showed a high concentration and purity compared with other fractions. The resultant fraction named LAP-1 was concentrated, collected, and freeze-dried for further research.

### 2.4 Characterization of LAP-1

#### 2.4.1 Physicochemical properties of LAP-1

The acidic carbohydrate content, total carbohydrate content and total protein content were measured by the *meta*-hydroxydiphenyl method,^10^ the phenol–sulfuric acid method^9^ and the Bradford method,^11^ respectively. Optical rotation and pH were determined by Autopol IV (Rudolph Research Instruments, Co., USA) and PB-10 (Sartorius, Germany). UV-2500PC (SHIMADZU Co., Japan) was used to measure the sample's ultraviolet spectrum in the scanning range of 200–700 nm and each measurement was repeated three times.

#### 2.4.2 Monosaccharide composition analysis

Trifluoroacetic acid (TFA) was used to hydrolyze the samples completely at 100 °C for 8 h.^12^ A stock standard solution was prepared by mixed each standard monosaccharide. Then, 50 μL sample solutions and 50 μL standard solutions were added to 50 mL of 0.6 mol L^−1^ NaOH. After mixing, a 100 mL PMP methanol solution was added at 70 °C for 30 min. After the reaction was completed, 100 μL 0.3 mol L^−1^ HCl was added to neutralize it. Distilled water and trichloromethane were used to extract the upper phase, repeated three times, and then the water phase was filtered through a 0.45 μm membrane. High-performance liquid chromatography (HPLC) coupled with a UV-detector and equipped with a ZORBAX Eclipse XDB-C18 (4.6 mm × 250 mm, 5 μm, Agilent, USA) was used to analyze the monosaccharide derivants at 250 nm. Potassium dihydrogen phosphate–acetonitrile (0.125 mol L^−1^) at a ratio of 84 : 16 was used as the mobile phase. The column temperature was set at 30 °C, and the flow rate was set at 1 mL min^−1^. The ratio was calculated by each monosaccharides standard by the above methods.

#### 2.4.3 Primary structure analysis

The IR spectrum of LAP-1 with KBr pellets between 400 and 4000 cm^−1^ was recorded by using a Nicolet-170X spectrophotometer (Nicolet Co., USA). An STA PT1600 synchronous thermal analyzer (Linseis, German) was used to test the thermal behavior of the samples. The sample weight was 5 mg; the gas was N_2_; and the heating rate was set at 5 °C min^−1^ from 30 °C to 800 °C, and Al_2_O_3_ was used as the reference. A Bruker AV-600 NMR spectrometer (Bruker, German) was used to determine the ^13^C NMR, ^1^H NMR and two-dimensional NMR spectra at 30 °C. The ^1^H–^1^H correlation spectrometry (COSY) was recorded in a phase-sensitive mode and the echo/antiecho gradient selection with decoupling was used to record the ^1^H–^13^C heteronuclear single-quantum coherence spectra (HSQC).

#### 2.4.4 Methylation and GC-MS analysis

NaBH_4_ and 1-ethyl-3-(3-dimethylaminopropyl)carbodiimide (EDC) was used to carboxyl-reduce LAP-1 according to the methods described in the previous literature.^13^ The complete methylation was verified by the FT-IR spectrum with O–H absorption band disappearance.

Then, formic acid (88%) and trifluoroacetic acid were used to hydrolyze the methylated samples for 6 h and 8 h at 100 °C, separately. After being reduced by NaBH_4_ and acetylated, the products were determined on a gas chromatography-mass spectrometer (6890N-5975B, Agilent, USA) that was equipped with an HP-5MS quartz capillary column (30 m × 0.25 mm × 0.25 μm). Temperature rising program: 1 °C min^−1^, 160–180 °C, 3 °C min^−1^ to 220 °C, held for 10 min. The mass charge ratio range was set at 25–500.

### 2.5 Additional structural analysis of LAP-1

#### 2.5.1 Molecular weight determination and multiangle laser light scattering analysis

Absolute molecular weight (*M*_w_), molecular weight distribution (*M*_w_/*M*_n_), and the molecular rotation radius (*R*_g_) of LAP-1 were measured by high performance size exclusion chromatography (Agilent 1260, Agilent, USA) coupled with an RI detector, a MALLs detector and a Shodex SUGAR SB-806 HQ column (Shodex, Tokyo, Japan) (Liu *et al.*, 2016).^26^ The dynamic light scattering analysis of the LAP-1 was detected by a DynaPro DLS System (Wyatt Technologies, USA). The scattering angle was 90°, and the temperature was 25 °C. Distilled water was the solvent and the flow rate was 1 mL min^−1^ and the refractive index was 1.330. The parameters were calculated from:4
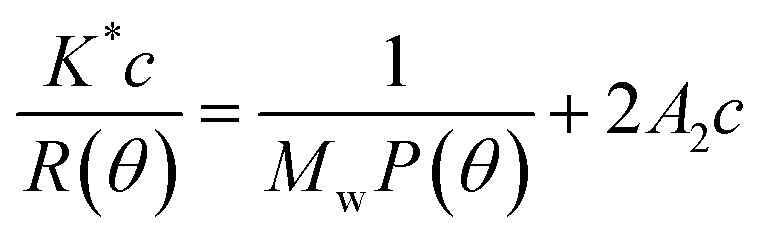
K*: 4π^2^(d*n*/d*c*)^2^*n*_0_^2^(*N*_A_*λ*_0_^4^), *N*_A_: Avogadro's number, *λ*_0_: wavelength, *n*_0_: refractive index of solvent, d*n*/d*c*: refractive index increment of polysaccharides, *A*_2_: second virial coefficient, *M*_w_: average molecular weight, *c*: concentration of polysaccharides (g mol^−1^), *R*(*θ*): uniangular splitting intensity of scattered light, *P*(*θ*): Rayleigh ratio.

#### 2.5.2 Congo red test and XRD analysis

Whether the conformation of LAP-1 was a triple helix was determined by Congo red analysis. Briefly, an LAP-1 solution (0.5 mg mL^−1^) was mixed with a sodium hydroxide solution at different concentrations (0.05, 0.1, 0.15, 0.2, 0.25, 0.3, 0.35, 0.4, 0.45, and 0.5 mol mL^−1^, respectively) and a Congo red solution (50 μg mL^−1^) at a volume ratio of 2 : 2 : 1. The control group included the same concentration of Congo red and a sodium hydroxide solution without LAP-1. After reaction for 10 min at room temperature, the absorbance was determined from 400 to 600 nm. A diffractometer Bruker AXS D8 (Bruker, Germany) was used to determine the crystalline characteristics of LAP-1. The scattering angles (2*θ*) were set at 5–80° with Ni-filtered Cu Kα radiation (*λ* = 1.5406 Å), and the current and voltage were set at 40 mA and 40 kV, respectively.

#### 2.5.3 Morphology analysis of LAP-1

Scanning electron microscopy (accelerating voltage: 15 kV) (JSM-7600F, JEOL, Japan), transmission electron microscopy (accelerating voltage: 80 kV) (JEM-2100, JEOL, Japan) and atomic force microscopy (Bruker, Germany) were used to explore the morphology and topography of LAP-1. Samples prepared for the SEM test were dried and deposited on metal stubs. The TEM test sample and the AFM sample were mounted on a carbon film and mica substrate, respectively.

### 2.6 Anticoagulant activity *in vitro*

Prothrombin time (PT), activated partial thromboplastin time (aPTT), and thrombin time (TT) of LAP-1 were evaluated by the method published with some modifications.^14^ Briefly, 20 μL sample solutions of different concentrations were mixed with 80 μL of citrated healthy human plasma (0.109 mol L^−1^ sodium citrate, ratio: 9 : 1, v/v). Then, 100 μL pretreated PT reagent, aPTT reagent and TT reagent were added to the mixture and incubated at 37 °C for 180 s. Clotting time was determined by an automatic coagulation analysis system (CS-5100, Sysmex, Japan). Normal saline (0.90%) and heparin sodium (0.002 mg mL^−1^) were used as the negative control and positive drug group, respectively.

### 2.7 Statistical analysis

All data are shown as the mean ± standard deviation (SD) of three determinations. The statistical analysis was calculated by using SPSS 22.0. *P* < 0.05 is defined as significant and *P* < 0.01 is defined as highly significant.

## 3. Results and discussion

### 3.1 Optimization of extraction conditions

#### 3.1.1 Effects of single factors

**Fig. 1:**
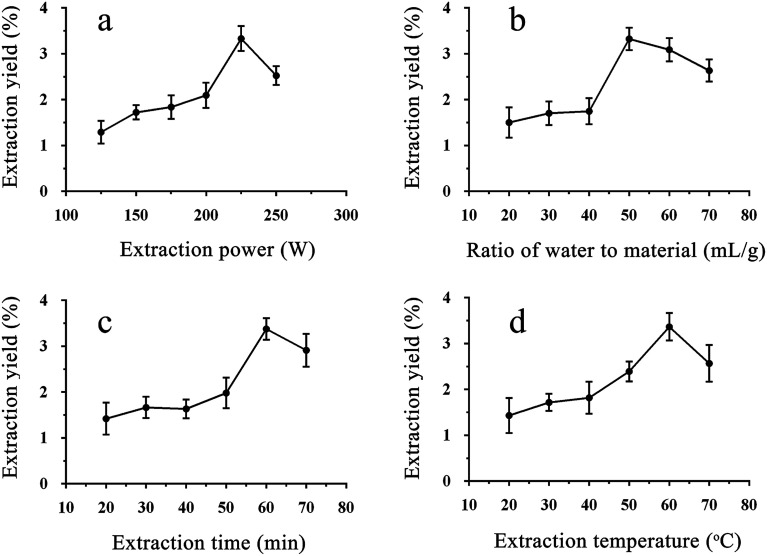
Effects of ultrasonic power (a), ratio of water to material (b), extraction time (c), and extraction temperature (d) on the extraction yield of crude polysaccharides. Each value represents mean ± SD of triplicates.

The effects of four extraction parameters were investigated in this study. Extraction power is a crucial parameter of ultrasonic-assisted extraction. As Fig. 1a shows, the yield increased with the extraction power but plateaued and then declined after 225 W. An appropriate extraction power ensures adequate cell disruption. The subsequent decrease may be explained by an overhigh ultrasonic power leading to the destruction of polysaccharides molecules.^8^ The extraction process was carried out under different ratios of water to materials. The yield increased and then declined as shown in Fig. 1b. We think that the sample powders probably could not disperse fully in inadequate solvents and superfluous water could reduce the thermal energy efficiency.

An appropriate temperature and time were beneficial for the yield of crude polysaccharides, while too much time and too high of a temperature had the opposite effect, as shown in Fig. 1c and d and the previous literature.^15^ The yield reached its peak at 60 °C and 60 min, and lower temperatures and shorter times made it hard to facilitate cell wall fragmentation.^15^ However, the curve decreased after the peak, and the reason for this could be that a higher temperature and too much time can damage the structure of crude polysaccharides due to the ultrasonic power.^16^

According to the above results, extraction powers of 200–250 W, extraction times of 50–70 min, extraction temperatures of 50–70 °C, and ratios of water to material from 40 : 1 to 60 : 1 were selected for the subsequent experiments.

#### 3.1.2 Optimization of extraction conditions by RSM

The experimental design and the corresponding experiments were carried out in triplicate as shown in Table 1. The following second-order polynomial formula was obtained from the analysis:

5 *Y* = 3.30 + 0.054*A* + 0.11*B* + 0.090*C* + 0.29*D* − 0.092*AB* − 0.11*AC* − 0.092*AD* + 0.060*BC* − 0.034*BD* − 0.020*CD* − 0.59*A*^2^ − 0.16*B*^2^ − 0.26*C*^2^ − 0.61*D*^2^

**Fig. 2:**
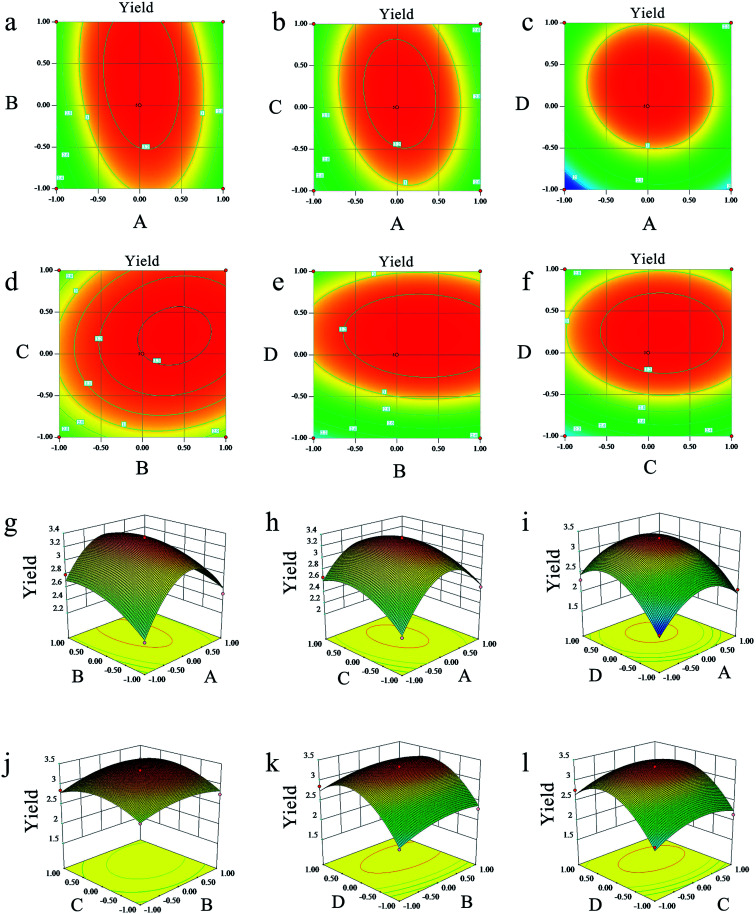
Response surface (3D) and contour plots (2D) display the influence factors (*A*: extraction time, min; *B*: ultrasonic power, W; and *C*: reaction temperature, °C; *D*: ratio of water to material, mL g^−1^) on the yield of crude polysaccharides.

As shown in Table 2, the *p*-value, determinant coefficient, and coefficient of variation were <0.01, 0.9734 and 5.95. These results showed that the polynomial model equation has good reliability and precision, that it can explain the relationship between the response value and the parameters exactly and that the quadratic terms have significant effects. The interaction of the dependent variable and other factors were illustrated by 2-D contours and 3-D response surfaces as shown in Fig. 2. While the ultrasonic power and the ratio stayed at a zero level, the response value increased with higher temperatures and longer times at the beginning and then declined. Other variables and response values showed similar situations.

**Table 2:** Analysis of variance of the experimental results of the BBD

Source	Coefficient estimate	Sum of squares	Degree of freedom	Standard error	Mean square	*F*-value	*P* value
Model	3.3	5.400	14	0.046	0.390	36.590	<0.0001^a^
*A*	0.054	0.035	1	0.030	0.035	3.280	0.092
*B*	0.11	0.150	1	0.030	0.150	14.270	0.002^a^
*C*	0.09	0.096	1	0.030	0.096	9.130	0.009^a^
*D*	0.29	1.020	1	0.030	1.020	97.060	<0.0001^a^
*AB*	−0.092	0.034	1	0.051	0.034	3.230	0.094
*AC*	−0.11	0.047	1	0.051	0.047	4.490	0.053
*AD*	−0.092	0.034	1	0.051	0.034	3.240	0.093
*BC*	0.06	0.014	1	0.051	0.014	1.370	0.262
*BD*	−0.034	0.005	1	0.051	0.005	0.430	0.522
*CD*	−0.02	0.002	1	0.051	0.002	0.150	0.703
*A* ^2^	−0.59	2.240	1	0.040	2.240	212.130	<0.0001^a^
*B* ^2^	−0.16	0.170	1	0.040	0.170	16.450	0.001^a^
*C* ^2^	−0.26	0.440	1	0.040	0.440	41.550	<0.0001^a^
*D* ^2^	−0.61	2.420	1	0.040	2.420	229.730	<0.0001^a^
Residual		0.150	14		0.011		
Lack of fit		0.140	10		0.014	5.950	0.052^b^
Pure error		0.009	4		0.002		
Cor total		5.550	28				
*R* ^2^		0.9734					
Adj *R*^2^		0.9486					
Pred *R*^2^		0.8538					
Adeq precision		22.160					
CV%		3.91					

a Means significance (significance level 0.01).

b Means significance (not significant).

The validation of this model was also proven by the predicted optimal experiments. The extraction conditions experiments showed that reaction temperature, reaction time, ultrasonic power and ratio of water to materials after response surface optimization were 62 °C, 60 min, 230 W, and 3.15 : 1, respectively. The extraction yield of LAP was 3.45 ± 0.27% under the predicted optimal conditions, which is consistent with the predicted optimal yield based on this model.

### 3.2 Characteristics of LAP-1

#### 3.2.1 Physicochemical properties and monosaccharide composition

**Fig. 3:**
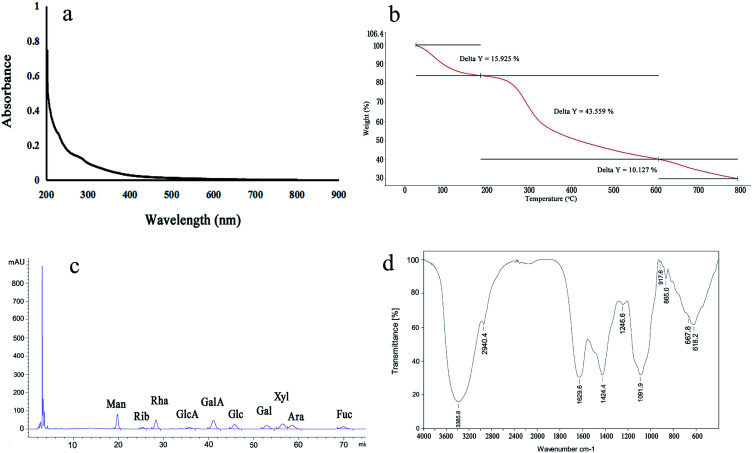
UV spectrum (a), TGA curves (b), monosaccharide composition (c) and IR spectrum (d) of LAP-1.

The acidic carbohydrate content, total carbohydrate content, optical rotation value and pH value were determined to be 10.39 ± 1.44%, 92.11 ± 4.97%, +19.0° and 6.30 ± 0.10, respectively. No nucleic acid or protein in the LAP-1 were detected by the Bradford method or the UV spectra, respectively (Fig. 3a). The thermal gravimetric results of LAP-1 are illustrated in Fig. 3b. In the heating progress, there were three stages that occurred at 50–200 °C, 200–600 °C and 600–800 °C, respectively. The structural and absorbed water loss caused by hydrogen bound water desorption that are typical characteristics of carbohydrate polymers can lead to the first weight decline and the maximum loss happening in the second stage.^16^

#### 3.2.2 Monosaccharide composition analysis

The monosaccharide composition of LAP-1 was analyzed by PMP-HPLC-UV. As shown in Fig. 3c, LAP-1 consisted of galacturonic acid (GalA), mannose (Man), xylose (Xyl), rhamnose (Rha), arabinose (Ara), glucose (Glc), galactose (Gal), fucose (Fuc), ribose (Rib), and glucuronic acid (GlcA) in molar ratios of 8.74 : 3.45 : 1.02 : 1 : 2.11 : 5.60 : 4.73 : 1.08 : 1.09 : 1.47. According to the results, LAP-1 showed a varied monosaccharide composition. The galacturonic acid, glucose, galactose were the major monosaccharides in LAP-1 which revealed that there were a large number of glycosidic linkages of galacturonic acid, glucose, galactose exist in LAP-1. Accurate glycosidic linkages identification was carried on follow-up experiments.

#### 3.2.3 FT-IR analysis

As shown in Fig. 3d, the deformation vibration and stretching vibration of C–H and stretching vibration of OH were verified by 2904.4 cm^−1^ weak peaks and a broad intense 3385.8 cm^−1^ peak, respectively.^5,17^ The peaks at 1629.6 cm^−1^ and 1424.0 cm^−1^ demonstrated the strong symmetric and asymmetric stretching characteristics caused by COO^−^ groups. The C–O–C stretching vibration and the C–H variable angle vibration of the saccharide ring were determined by the bands approximately 1246.6 cm^−1^ and 1091.9 cm^−1^. Furthermore, the β-d-pyranosidic and α-d-pyranosidic structure of LAP-1 that were proven by the optical rotation values above were consistent with the peaks approximately 865.0 cm^−1^ and 917.6 cm^−1^.

#### 3.2.4 Methylation-GC-MS analysis

The further features of the glycosidic linkages of LAP-1 were investigated by methylation and GC-MS analysis. The results demonstrated that nonreducing terminal Glc*p*, nonreducing terminal Man*p*, 1,2-linked-Ara*p*, 1,3-linked-Gal*p*, 1,4-linked-Man*p*, 1,4-linked-Gal*p*, 1,4-linked-Glc*p*, 1,4-linked-GalA*p*, 1,4-linked-GlcA*p*, 1,4,6-linked-Man*p*, and 1,3,4-linked-Man*p* existed in LAP-1. According to the physicochemical properties, monosaccharide composition and FT-IR analysis, it could be concluded that LAP-1 has a typical pectin structure.^18^ There are several domains that exist in pectin structures such as xylogalacturonan (XGA) regions, branched rhamnogalacturonan (RG-I and RG-II) as well as linear homogalacturonan (HG) regions. For further description of the detailed structure of *p*-LAP-1, NMR was carried out.

#### 3.2.5 NMR analysis

**Fig. 4:**
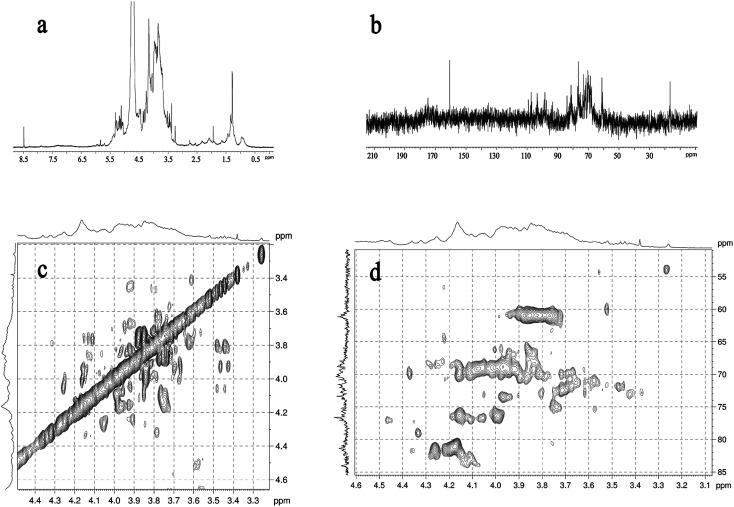
NMR spectrum of LAP-1: (a) ^1^H NMR spectrum; (b) ^13^C NMR spectrum; (c) ^1^H–^1^H COSY spectrum; (d) HSQC spectrum.

The main chemical shifts of the LAP-1 are shown in Fig. 4. The signals of LAP-1 were in the 3.0–5.5 ppm regions (^1^H NMR, Fig. 4a) and the 60–110 ppm regions (^13^C NMR, Fig. 4b). After analyzing the 2D-NMR spectrum, the HSQC spectrum and the data from other related literature, the signals of LAP-1 are summarized in Table 3. The results indicated that characteristic linkages of polysaccharides such as →1)-α-d-Man*p*, →1)-α-d-Glc*p*, →1)-α-d-Ara*p*-(2→, →1)-β-d-Gal*p*-(3→, →1)-β-d-Man*p*-(4→, →1)-β-d-Gal*p*-(4→, →1)-β-d-Glc*p*-(4→, →1)-β-d-GalA*p*-(4→, →1)-β-d-GlcA*p*-(4→, →1)-β-d-Man*p*-(4,6→, →1)-β-d-Man*p*-(3,4→ existed in LAP-1 and these results were consistent with the GC-MS analysis. Based on the methylation analysis and NMR spectra results, LAP-1 contains characteristic linkages features of pectin.^19,20^ According to the previous literature, pectins show diverse activities including anticoagulant activity, immunomodulation, reducing proliferation and inducing apoptosis,^21^ and these activities are based on their characteristic structural features.^22,23^

**Table 3:** Chemical shifts for the resonances of glycosyl residues of LAP-1 in NMR spectra

Glycosyl residues	*δ* ^13^C/^1^H (ppm)					
	C1	C2	C3	C4	C5	C6
	H1	H2	H3	H4	H5	H6
→1)-α-d-Man*p*	93.93	71.59	70.84	67.99	72.79	61.04
	5.32	3.94	3.85	3.68	3.81	3.74/3.86
→1)-α-d-Glc*p*	93.47	72.04	73.78	70.40	72.02	61.46
	5.29	3.53	3.71	3.42	3.83	3.76/3.80
→1)-α-d-Ara*p*-(2→	98.48	75.23	76.71	70.07	65.99	
	5.22	3.26	3.43	3.63	3.33	
→1)-β-d-Gal*p*-(3→	103.26	70.37	84.01	70.00	75.69	61.35
	4.49	3.75	3.77	4.10	3.69	3.68/3.74
→1)-β-d-Man*p*-(4→	103.52	73.39	75.23	80.33	75.69	61.04
	4.58	3.42	3.66	3.63	3.59	3.83/3.96
→1)-β-d-Gal*p*-(4→	107.54	70.37	73.80	81.02	75.69	61.35
	4.49	3.75	3.77	4.16	3.69	3.68/3.74
→1)-β-d-Glc*p*-(4→	109.29	72.04	73.78	81.36	72.02	61.46
	5.09	3.44	3.67	3.60	3.59	3.83/3.96
→1)-β-d-GalA*p*-(4→	107.54	70.37	73.80	81.02	75.69	160.77
	4.49	3.75	3.77	4.16	3.69	8.48
→1)-β-d-GlcA*p*-(4→	109.29	72.04	73.78	81.36	72.02	160.77
	5.06	3.44	3.67	3.60	3.59	8.48
→1)-β-d-Man*p*-(4,6→	109.36	71.59	76.85	84.11	72.79	68.81
	5.15	3.46	3.88	4.45	3.52	4.06/3.65
→1)-β-d-Man*p*-(3,4→	109.37	70.37	81.45	81.78	72.79	60.88
	5.12	3.48	4.05	4.35	3.50	3.58/3.45

### 3.3 Additional structural analysis of LAP-1

#### 3.3.1 Results of HPSEC-MALLS-RID and dynamic light scattering

**Fig. 5:**
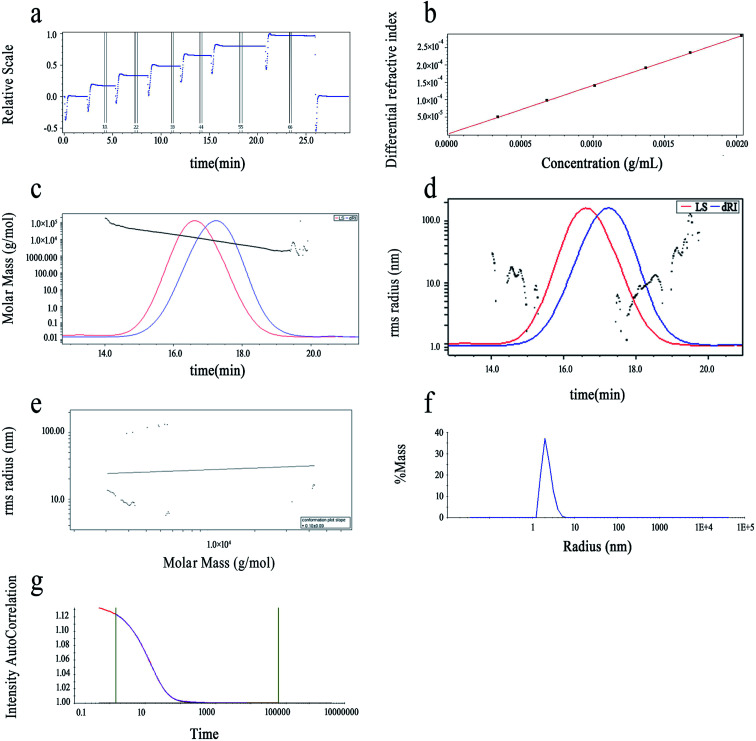
HPSEC-MALLS chromatogram of LAP-1: (a and b) d*n*/d*c* determination of LAP-1; (c) HPSEC elution curve of LAP-1; (d) *R*_g_ distribution of LAP-1; (e) dependence of *R*_g_ on *M*_w_ for LAP-1; (f) the distribution diagram of hydrodynamic radium (*R*_h_) of LAP-1 in the deionized water by dynamic light scattering detector; (g) the intensity–intensity time correlation function of LAP-1 in self-beating mode.

Chain conformation, molecular weight and spatial configuration are three essential parameters for pectin's biological activities. Light scattering is usually used to determine polysaccharides' absolute molecular weights, which are related to their biological activities. A precise molar mass measurement depends on the particle scattering. The solvent-substance system's refractive index increment (d*n*/d*c*) is a crucial factor for particle scattering.^24^ In addition, HPSEC-MALLs-RID can measure the corresponding average radius of gyration (*R*_g_), polydispersity (*M*_w_/*M*_n_), number average molecular weight (*M*_n_), *z*-average molecular weight (*M*_*z*_), weight-average molecular weight (*M*_w_) and molecular conformation, branching information, and distributions of the molecular weight of the polymers.^25^ As Fig. 5a and b show, the d*n*/d*c* of LAP-1 was determined to be 0.1380 (±0.5429%) mL g^−1^.

The purity of LAP-1 was verified by a relatively sharp symmetric peak as shown in Fig. 5c. The *M*_w_/*M*_n_ (PDI), *M*_n_, *M*_*z*_ and *M*_w_ of LAP-1 were measured to be 1.423, 6.979 × 10^3^ g mol^−1^, 1.409 × 10^4^ g mol^−1^, and 9.930 × 10^3^ g mol^−1^. As Fig. 5d indicates, the 〈*s*^2^〉 *z*^1/2^ (*R*_g_) of LAP-1 is 7.1 (±1.2%) nm. The instability of the RMS radius curves are caused by the compact branched molecules of LAP-1 and chain entanglement between large molecules and small molecules affecting the co-eluting time.

The molecular conformation and molecular size of polymers can be determined by index *ν* and the root-mean-square (RMS) radius 〈*s*^2^〉 *z*^1/2^ (*R*_g_).^26^ Branching, sphere, flexible random coil conformation, and rigid rod correspond to the *ν* values of 0.1–0.4, 0.3, 0.5–0.6, 0.6–1.0, respectively.^27^ As calculated from Fig. 5e, the *ν* of LAP-1 was 0.10, *κ* was 2.83, and the df was 10, indicating that a high branch structure existed in LAP-1.

The chain conformation and structure of the LAP-1 molecule could be measured by its *ρ* value.^28^ The *ρ* value was calculated through the equation: *ρ* = *R*_g_/*R*_h_ and *R*_h_, *R*_g_ determined by DLS and SLS, respectively. The *ρ* values of ≥2, 1.5–1.8 and 0.775 represent extended chains and flexible random coils and spheres, respectively.^25^ As shown in Fig. 5f and g, the *ρ* value, *R*_h_ and *R*_g_ of LAP-1 were 3.09, 2.3 nm, and 7.1 nm. Thus, LAP-1 contains a high branch structure according to the *ρ* value and the results were consistent with the LLS.

#### 3.3.2 Congo red test and XRD analysis

**Fig. 6:**
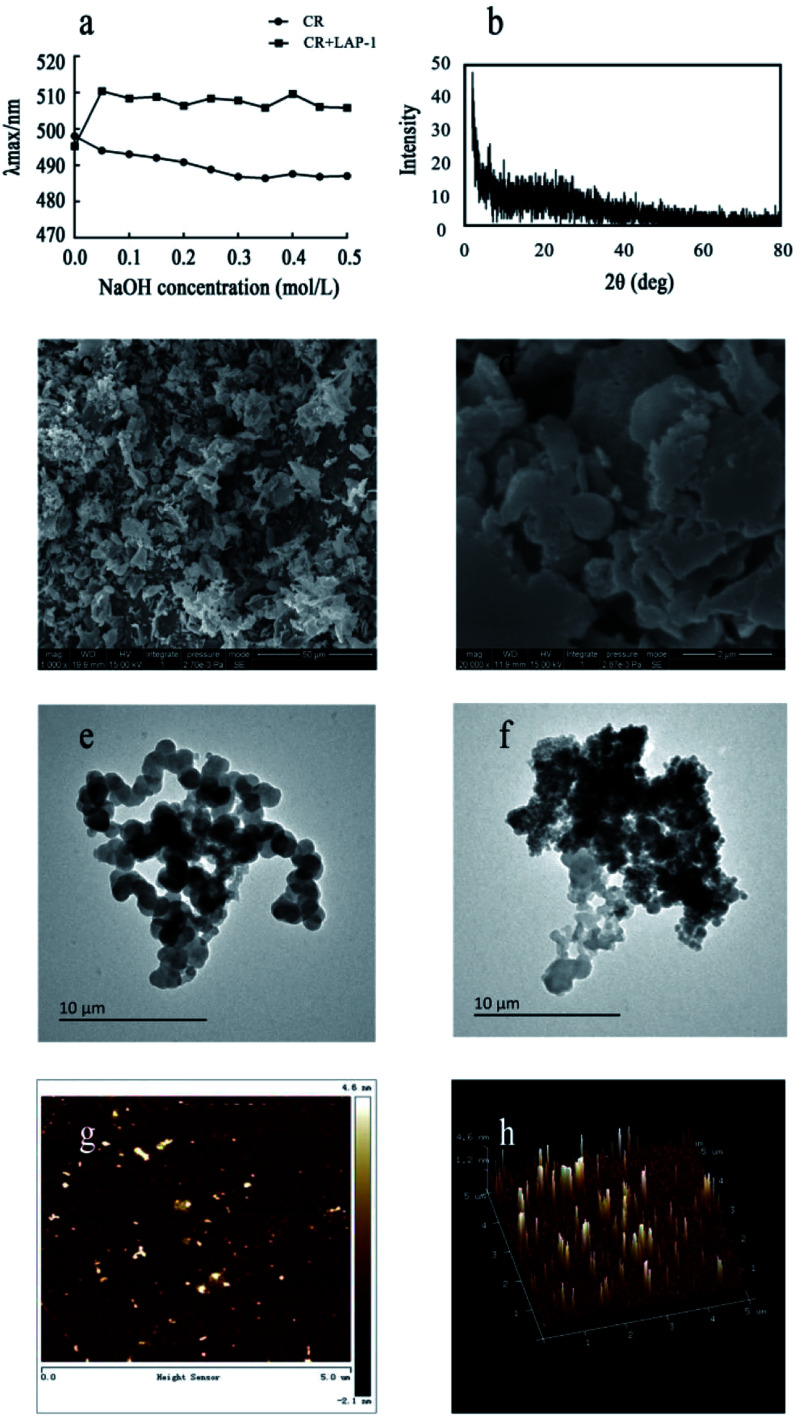
Additional structural analysis of LAP-1: (a) maximum absorption (*λ*_max_) of Congo red and Congo red + LAP-1 at various NaOH concentrations; (b) XRD spectra of LAP-1; (c and d) the SEM images of LAP-1 in the deionized water, scale bar is 50 μm and 2 μm, respectively; (e and f) the TEM images of LAP-1 in the deionized water, scale bar is 10 μm; (g) atomic force microscope planar image; (h) three-dimension image of atomic force microscope.

The spatial stereochemical structure of the polysaccharides can be detected by the wavelength changes of the Congo red experiment. At present, the common method is to use the Congo red test to detect whether there is a triple helix structure in the polysaccharide solution. Congo red is an acidic dye that can dissolve in water and alcohol. It can form a complex with polysaccharides with three helical conformations. The maximum absorption wavelength of the complex is redshifted compared with Congo red. Within a certain concentration range, the characteristic change of the maximum absorption wavelength becomes purple red. When the NaOH concentration is higher than a certain value, the maximum absorption wavelength decreases sharply.^29^ As Fig. 6a shows, the LAP-1 group and the control group have the same maximum absorption wavelengths without the redshifted phenomenon appearance. These results indicated that there were no triple helical conformations existing in LAP-1.

X-ray diffraction (XRD) is a rapid and highly effective method to obtain information about the composition of materials and the structure or morphology of atoms or molecules in materials. The crystal structure causes the incident X-ray beam to diffract in many specific directions. By measuring the angle and intensity of these diffracted beams, crystallographers can generate three-dimensional images of the electron density in crystals. Based on this electron density, the average position of atoms in the crystal, their chemical bonds and various other information can be determined. As Fig. 6b shows, there were no diffraction peaks in the spectra, indicating that LAP-1 is not amorphous in nature. Multifarious spatial structures of pectins with various biological activities exist and a crystalline structure or a triple helical structure are the only two types.

#### 3.3.3 Molecular morphology

Scanning electron microscopy (SEM), transmission electron microscopy (TEM) and atomic force microscopy (AFM) were used to find visual evidence of LAP-1's chain conformation. SEM is a microscopic morphology observation method between TEM and optical microscopy (OM) that can directly use the material properties of the appearance of a polysaccharide surface for microscopic imaging.^30^ As shown in Fig. 6c and d, the surface of LAP-1 was amorphous in structure and had a relatively rough surface. TEM accelerates and aggregates electron beams onto thin samples. Electrons collide with atoms in the samples to change direction, resulting in stereoangular scattering. The scattering angle is related to the density and thickness of the sample, so different images can be formed. TEM is only used to observe ultrastructure that is less than 0.2 microns and cannot be seen under an optical microscope. It is also called the “submicroscopic structure”.^31^ As shown in Fig. 6e and f, LAP-1 showed an aggregation form with branches caused by the van der Waals' forces and the hydrogen bonds.

AFM can detect the physical properties of various materials and samples in the nanoregion, including morphology, in the atmosphere and liquid environment, or directly manipulate the nanoregion. It has been widely used for semiconductors, nanofunctional materials, biology, the chemical industry, food, pharmaceutical research and research into various nano-related disciplines and has become a basic tool for nanoscientific research.^32^ Herein, as shown in Fig. 6g and h, the majority of LAP-1 has a nebula-like structure with a particles aggregation form and scattered on a plane. The height, width and length of the surface typology of LAP-1 were 0.2–4.6 nm, 100–300 nm, and 0.1–2 μm, respectively while the normal size of polysaccharide chains is approximately 1.0 nm. The chain size of LAP-1 indicated that a particular aggregation occurred in LAP-1.^33,34^ Aggregation of polysaccharides occurs from their side branches entangling each other, and the substitution of the hydroxyl group of polysaccharides can could twisting and converting of the sugar ring conformation, which leads to a decrease of the size of the resulting polysaccharides. The aggregation of polysaccharides could related to some biological activities.

### 3.4 Anticoagulant activity *in vitro*

Different stages of the coagulation process can be demonstrated by APTT, TT and PT assays *in vitro*.^35^ The coagulation process can be divided into an endogenous coagulation pathway, an exogenous coagulation pathway and a common coagulation pathway.^36^ APTT assays use brain lipids and activators instead of platelets to detect VIII, IX, XI and excitatory releasing enzymes in the endogenous coagulation pathway to reflect the effects of endogenous factors on coagulation time.^37^ PT assays are conducted by adding thromboplastin to plasma to reflect the effect of exogenous factors on coagulation time. TT is a simple screening test for fibrin polymerization. It measures the formation time of fibrin from fibrinogen after adding a certain amount of thrombin to plasma.^38^ The results of the tests of anticoagulant activity of LAP and LAP-1 are summarized in Table 4. Both LAP and LAP-1 showed concentration-dependent anticoagulant activity under the range of experimental concentrations. LAP-1 showed the highest anticoagulant activity at 4 mg mL^−1^, which had a significant difference from the control groups (*P* < 0.01 or *P* < 0.05), while LAP at 4 mg mL^−1^ demonstrated a smaller difference than saline. A possible explanation for this could be related to the content of sugar and the purity of the polysaccharides. Furthermore, compared with the control group, the APTT, PT and TT increased 1.24-fold, 1.04-fold and 1.14-fold with the addition of LAP-1 at 4 mg mL^−1^, and the blood clotting time was prolonged to 33.85 s. Based on the above results, the anticoagulant activity of LAP-1 was mediated through inhibiting thrombin activity, preventing the conversion of fibrinogen to fibrin, and inhibiting the intrinsic/common pathways.^39,40^ Compared with the traditional anticoagulant drug heparin sodium, LAP-1 could inhibit blood coagulation through the exogenous and endogenous coagulation pathways with mild efficacy, low toxicity, less spontaneous bleeding, and no other adverse reactions.

**Table 4:** Analysis of the anticoagulant activities of the LAP-1 and LAP as measured by the APTT, PT and TT^a^

Sample	Concentration (mg mL^−1^)	APTT(s)	PT(s)	TT(s)
Saline	0.90%	27.21 ± 1.52	14.26 ± 0.28	11.20 ± 0.20
Heparin	0.002	33.32 ± 1.21**	14.87 ± 0.24*	12.77 ± 0.19**
LAP-1	0.125	27.51 ± 1.44	14.46 ± 0.47	11.74 ± 0.51
	0.25	28.36 ± 1.47	14.48 ± 0.50	11.90 ± 0.41*
	0.5	29.94 ± 0.63*	14.55 ± 0.37	12.01 ± 0.22**
	1	31.06 ± 1.00*	14.61 ± 0.52	12.16 ± 0.21**
	2	32.18 ± 1.87*	14.65 ± 0.33	12.35 ± 0.46**
	4	33.85 ± 1.48**	14.84 ± 0.28*	12.73 ± 0.55**
LAP	0.125	27.32 ± 1.50	14.01 ± 0.96	11.51 ± 0.29
	0.25	27.74 ± 0.69	14.00 ± 0.02	11.52 ± 0.29
	0.5	27.82 ± 0.67	14.03 ± 0.73	11.56 ± 0.12
	1	28.10 ± 1.65	14.08 ± 0.76	11.56 ± 0.25
	2	28.83 ± 0.99	14.23 ± 0.40	11.53 ± 0.61
	4	29.38 ± 0.80*	14.60 ± 0.45	11.80 ± 0.31*

a Data in the table were expressed as mean ± SD (*n* = 3 in each group). *Significantly inhibited compared with the saline group (*P* < 0.05). **Significantly inhibited compared with the saline group (*P* < 0.01).

In previous studies, the anticoagulant activity of polysaccharides was related to their acid sugar, sulfur structures and aggregation form.^35,37,39^ LAP-1 contained a higher ratio of galacturonic acid and a large number of →1)-β-d-GalA*p*-(4→ and →1)-β-d-GlcA*p*-(4→ compared with other polysaccharides and the spatial stereochemical structure of LAP-1 showed an aggregation form with branches caused by the van der Waals' forces and the hydrogen bonds which different with other plant carbohydrate polymers. The coagulation activity of LAP-1 could attributes to its low molecular weight, glycosidic linkages of galacturonic acid and aggregation form according to the results.

Anticoagulants can be used to prevent endovascular embolism or thrombosis, stroke or other thrombotic diseases by affecting certain coagulation factors during the process of coagulation.^41^ Healthy bodies have effective blood coagulation, anticoagulation and fibrinolysis systems, so blood does not coagulate or bleed from the blood vessels, and it always flows freely to complete its function.^42^ However, when the body is in a high coagulation state or when anticoagulation and fibrinolysis are weakened, thromboembolic diseases occur.^43^

Thrombosis often causes local blood coagulation in the vascular system, leading to health-related diseases including strokes and heart attacks.^44^ Risk factors for thrombosis include abnormal hyperlipidemia, hyperglycemia elevated blood pressure and cancer.^45^ These thrombotic diseases have developed into the main causes of death, so effective anticoagulant drugs are urgently needed. Based on the above data analysis, it can be concluded that LAP-1 has a certain anticoagulant effect *in vitro* and could be developed as an anticoagulant drug and applied to the treatment of coagulation-related diseases.

## 4. Conclusions

In this study, a novel pure bioactive polysaccharide was isolated and purified from *Leonurus artemisia* (LAP-1). The structural characteristics and bioactivities of LAP-1 was under a systematic exploration. Results from this study suggested that LAP-1 (*M*_w_: 9.930 × 10^3^ g mol^−1^) was mainly composed of galacturonic acid (GalA), mannose (Man), xylose (Xyl), rhamnose (Rha), arabinose (Ara), glucose (Glc), galactose (Gal), fucose (Fuc), ribose (Rib), and glucuronic acid (GlcA). According to IR spectrum, methylation analysis and NMR spectra, LAP-1 was composed of →1)-α-d-Man*p*, →1)-α-d-Glc*p*, →1)-α-d-Ara*p*-(2→, →1)-β-d-Gal*p*-(3→, →1)-β-d-Man*p*-(4→, →1)-β-d-Gal*p*-(4→, →1)-β-d-Glc*p*-(4→, →1)-β-d-GalA*p*-(4→, →1)-β-d-GlcA*p*-(4→, →1)-β-d-Man*p*-(4,6→, →1)-β-d-Man*p*-(3,4→. Besides, LAP-1 had a high branch structure measured by MALLS, SEM, TEM, and AFM. In addition, LAP-1 showed an obvious anticoagulant activity with low toxicity compared with the traditional anticoagulant drug heparin sodium. These experimental results provide chemical and bioactive fundamental for the development of LAP-1 as a potential anticoagulant drug and are worth further research.

## Conflicts of interest

The authors declare no conflict of interest.

## Supplementary Material
